# Supplementary material for: The Large and Diverse Family of Mediterranean Flat Breads: A Database
Source: Foods. 2022 Aug 4;11(15):2326. doi: 10.3390/foods11152326 (PMC9368210; doi:10.3390/foods11152326)
Supplement: Supplementary file 1 [file foods-11-02326-s001.zip › foods-1835792-supplementary.pdf]

Supplementary Table S1. List of questions for interviewing the bakers on flat bread production.

Product
What is the most common name of this flat bread? Please specify if you know also other names.
Is the production artisanal or industrial? (This question aims to understand how important the contribution of practical experience and manual skills is in the production of flat bread)
Is this flat bread produced throughout the entire country or is typical of a single town or region? (In the latter case specify the name of town or region)
In case this flat bread is produced in a single geographic area, is it marketed also outside that area?
What is the thickness of the flat bread? What is its diameter? What is its weight?
What are the main quality features of the flat bread? (Flexibility, uniformity of color, regularity of shape…)
What is the shelf life of the product?
Is the product packaged? How? (Which material? Under modified atmosphere? If yes which type?)*
What are the storage conditions?*
Process
What's the recipe? (Only flour, water, yeast and salt or also other ingredients? Possibly give the ratios)
What kind of flour is used (Whole grain or refined? From durum wheat, soft wheat, or other cereals or non-cereal grains?)
What are the quality criteria for selecting the raw material, if known? (Protein content, gluten content, gluten quality indices)
Is yeast used?
What kind of yeast is used? (Commercial bakers' yeast, chemical yeast, sourdough?)
If fats are included in the dough, what type of fats are used? (For example lard or oil)
Which kneader is used?
What is the time of kneading?
What are time and temperature of leavening?
How is shaping conducted? (Manually or mechanically?)
Is there a second leavening? (Time and temperature?)
What kind of oven is used? (Modern gas / electric oven or old-style wooden fueled oven? Is any traditional baking system used, such as "testo", "tannur" etc.?)
Could you specify time and temperature of cooking?
Is a second cooking performed to dry bread? What are time and temperature?
Cultural and ethnographic features
Is there any relationship between this bread and a specific religious celebration or an important time of the year for any rural tradition (harvest, sowing, etc.)?

*Questions removed after consulting with the President of a consortium of bakers specialized in the production of flat bread, because were considered too technical.
